# Supplementary material for: Enhancement of the Antitumor and Antimetastatic Effect of Topotecan and Normalization of Blood Counts in Mice with Lewis Carcinoma by Tdp1 Inhibitors—New Usnic Acid Derivatives
Source: Int J Mol Sci. 2024 Jan 19;25(2):1210. doi: 10.3390/ijms25021210 (PMC10816808; doi:10.3390/ijms25021210)
Supplement: Supplementary file 1 [file ijms-25-01210-s001.zip › Supplement 09.01.24.pdf]

Supplementary materials

Enhancement of the antitumor and antimetastatic effect of topotecan and normalization of blood counts in mice with Lewis carcinoma by Tdp1 inhibitors - new usnic acid derivatives

Tatyana E. Kornienko[1#], Arina A. Chepanova[1#],Alexandra L. Zakharenko[1*], Aleksandr S. Filimonov[2], Olga A. Luzina[2], Nadezhda S. Dyrkheeva[1], Valeriy P. Nikolin[3], Nelly A. Popova[3], Nariman F. Salakhutdinov[2], Olga I. Lavrik[1]

[1] Novosibirsk Institute of Chemical Biology and Fundamental Medicine, Siberian Branch of the Russian Academy of Sciences, 8, Akademika Lavrentieva Ave., Novosibirsk 630090, Russian Federation
[2] N. N. Vorozhtsov Novosibirsk Institute of Organic Chemistry, Siberian Branch of the Russian Academy of Sciences, 9, Akademika Lavrentieva Ave., Novosibirsk 630090, Russian Federation
[3] Institute of Cytology and Genetics, 10, Akademika Lavrentieva Ave., 630090 Novosibirsk, Russian Federation
* Correspondence: a.zakharenko73@gmail.com
\# The authors contributed equally

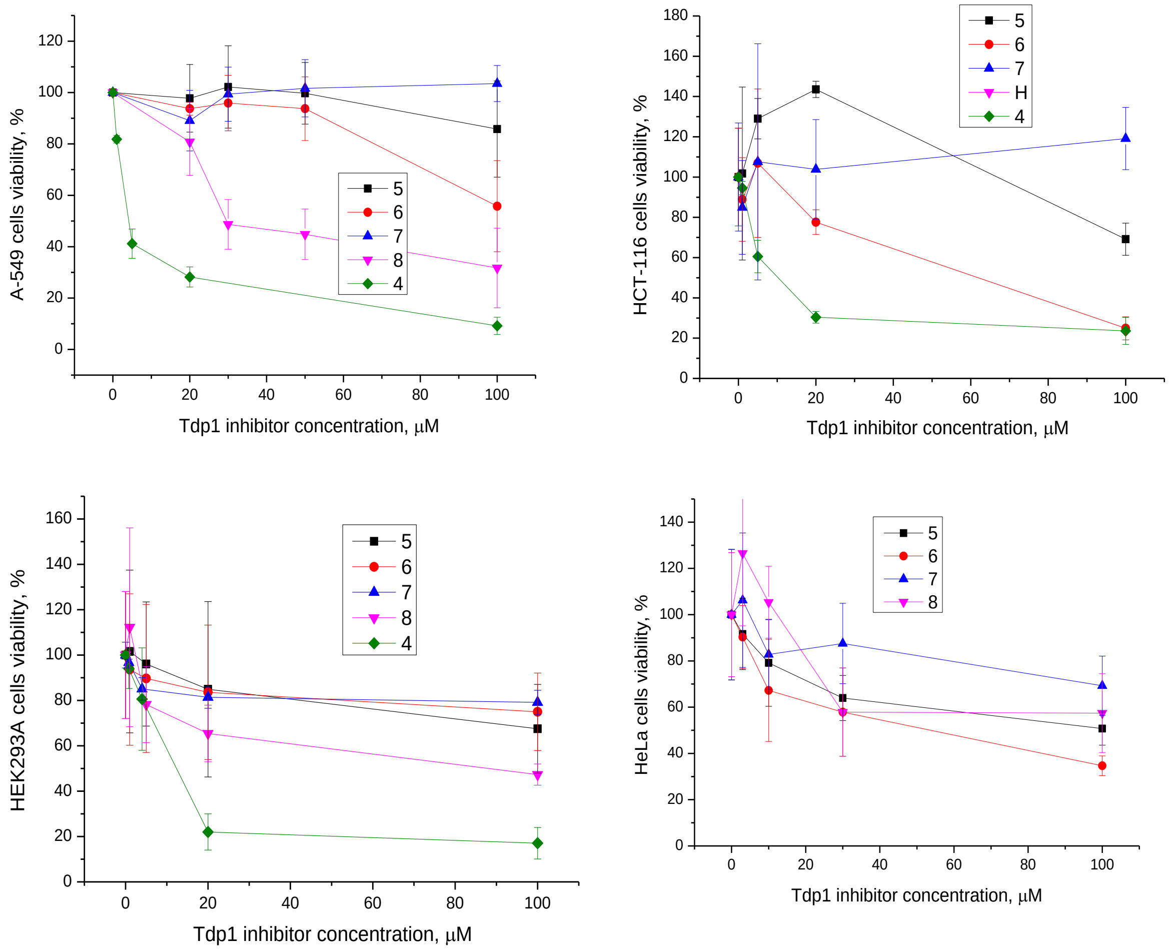

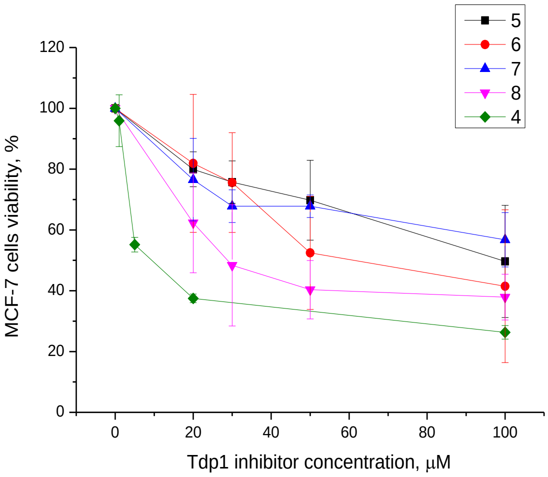

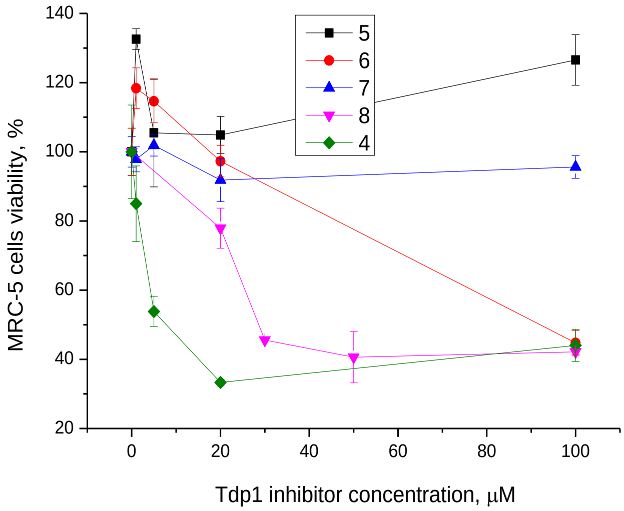

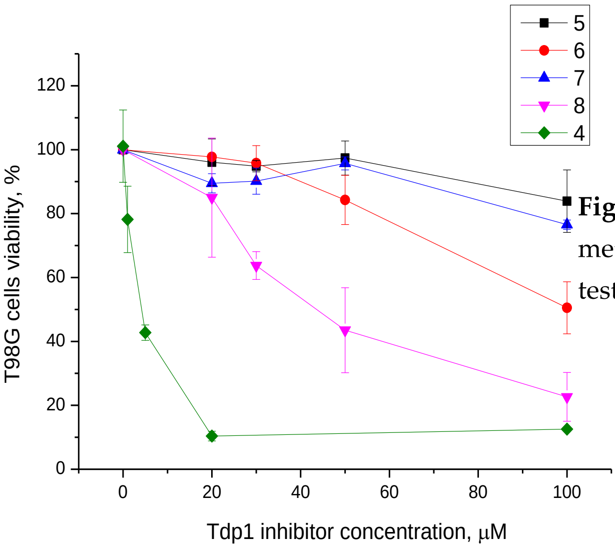

Figure S1. The effect of the compounds on the metabolic activity of cells according to the MTT test. Typical graphs. Values ± SEM are given

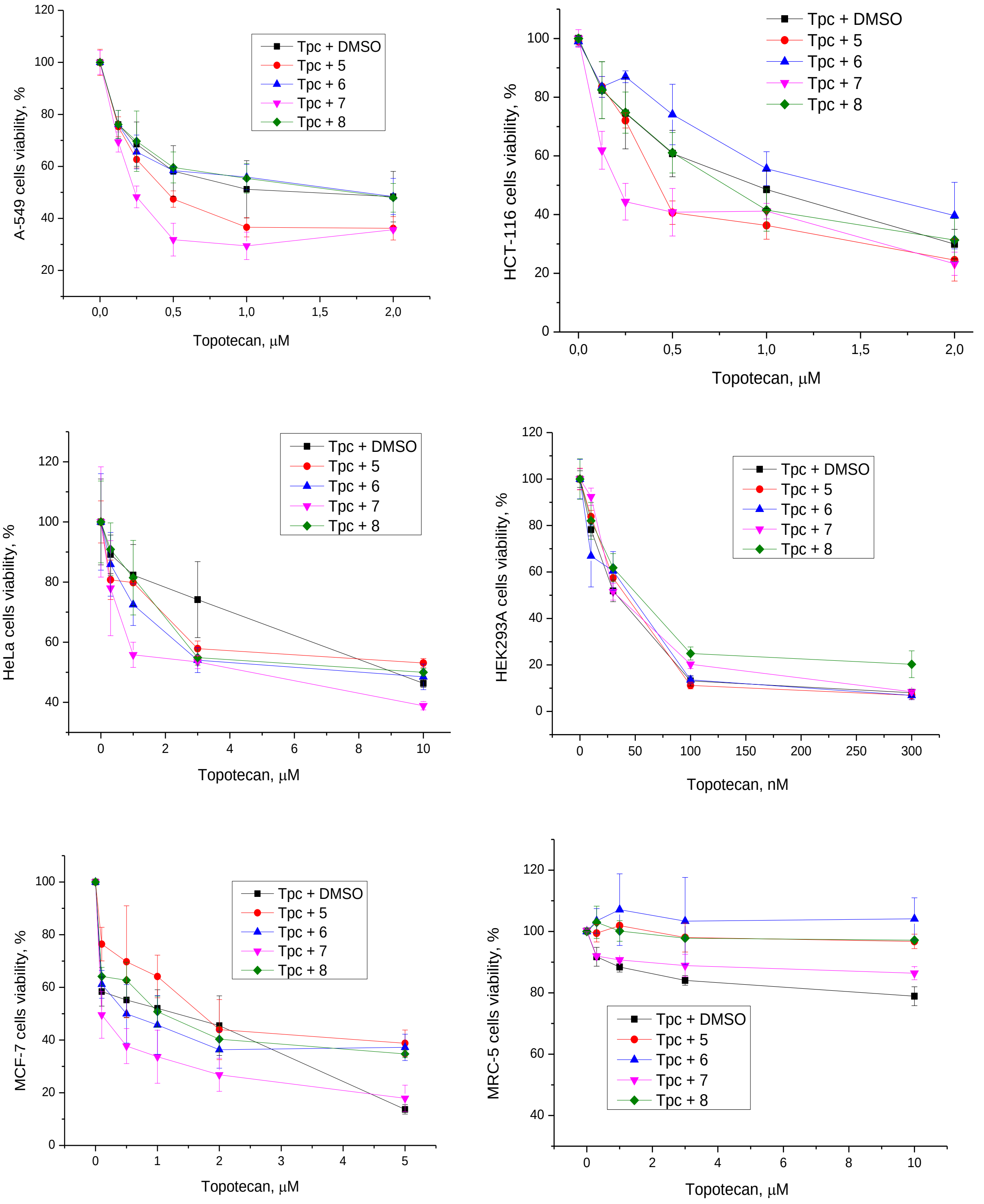

A-549 cells viability, %
Topotecan, µM
Tpc + DMSO
Tpc + 5
Tpc + 6
Tpc + 7
Tpc + 8
HCT-116 cells viability, %
Topotecan, µM
Tpc + DMSO
Tpc + 5
Tpc + 6
Tpc + 7
Tpc + 8
HeLa cells viability, %
Topotecan, µM
Tpc + DMSO
Tpc + 5
Tpc + 6
Tpc + 7
Tpc + 8
HEK293A cells viability, %
Topotecan, nM
Tpc + DMSO
Tpc + 5
Tpc + 6
Tpc + 7
Tpc + 8
MCF-7 cells viability, %
Topotecan, µM
Tpc + DMSO
Tpc + 5
Tpc + 6
Tpc + 7
Tpc + 8
MRC-5 cells viability, %
Topotecan, µM
Tpc + DMSO
Tpc + 5
Tpc + 6
Tpc + 7
Tpc + 8

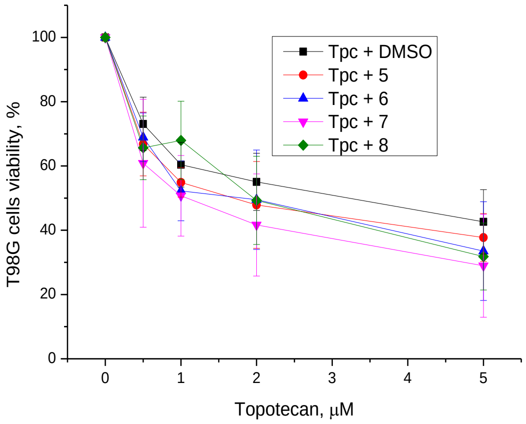

Figure S2. Effect of compounds on the cytotoxic/antiproliferative effect of topotecan.

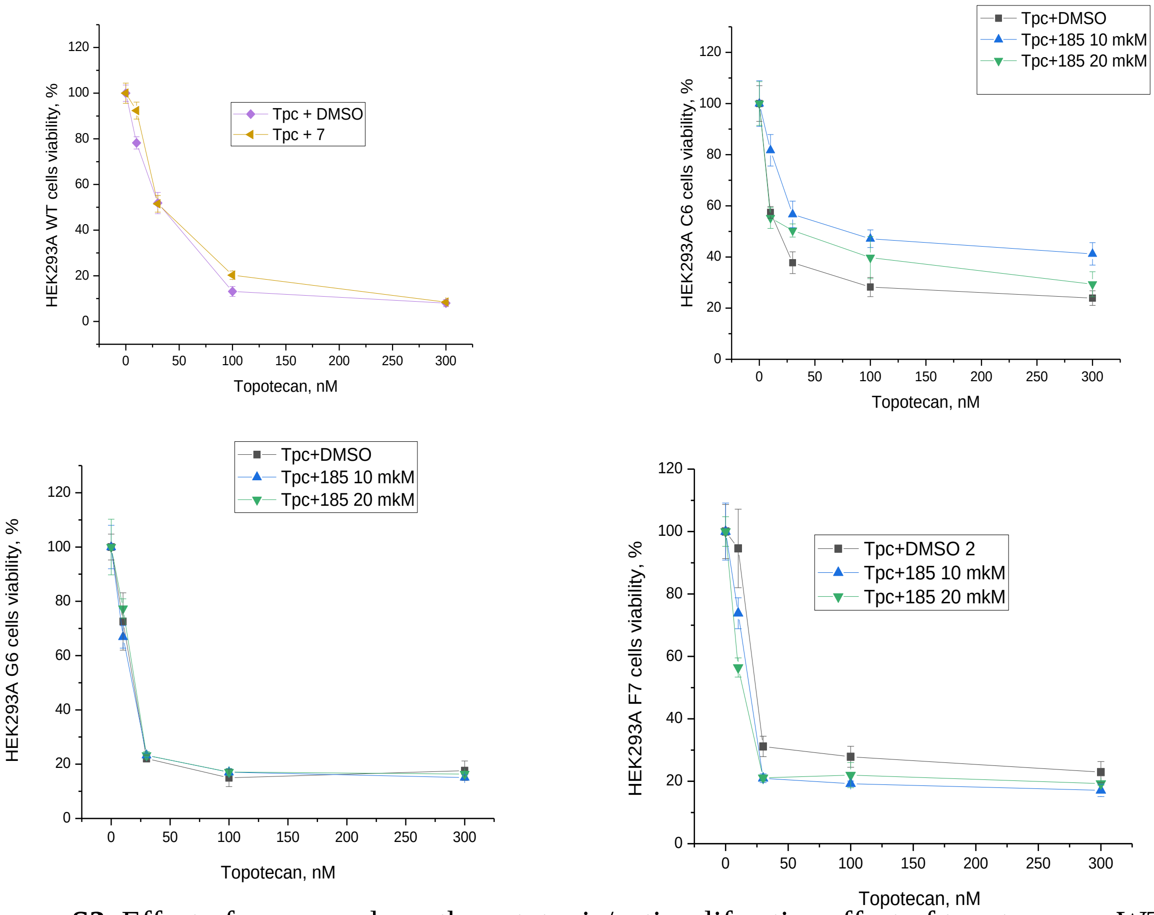

Figure S3. Effect of compounds on the cytotoxic/antiproliferative effect of topotecan on WT and Tdp1-/- (C6, G6, F7) HEK293A cells

Table S1. The organ indexes (%) of mice with Lewis lung carcinoma

Organ	Intact control	DMSO+Tween-80 i/g	Topotecan	Topotecan +**7** i/g	Topotecan +**7** i/p	**7** i/p	**7** i/g
Liver	5.0±0.4	5.2±0.3	5.6±0.7	4.9±0.7	4.9±0.2	4.6±0.5	4.9±0.3
Spleen	1.3±0.2	1.3±0.2	1.19±0.04	1.2±0.2	1.4±0.2	1.3±0.2	1.3±0.2

Table S2. The organ indexes (%) of mice with Krebs-2 carcinoma

Organ	Intact control	DMSO + Tween-80 i/g	DMSO + Tween-80 i/p	Topotecan	Topotecan +**7** i/g	Topotecan +**7** i/p	**7** i/p	**7** i/g
Liver	4.3±0.6	4.7±0.5	4.1±0.7	4.8±0.6	4.8±0.5	4.3±0.3	4.1±0.3	4.4±0.5
Spleen	1.2±0.2	1.35±0.09	1.2±0.3	1.3±0.2	1.3±0.2	1.2±0.1	1.0±0.1	1.2±0.1
